# Enhancing employee retention by promoting decent work in a South African financial services institution

**DOI:** 10.3389/fsoc.2026.1835488

**Published:** 2026-07-10

**Authors:** Patricia Lungile Setshogoe, Wilfred Isioma Ukpere, Musawenkosi Donia Saurombe

**Affiliations:** College of Business and Economics, Department of Industrial Psychology and People Management, University of Johannesburg, Johannesburg, South Africa

**Keywords:** casual employment, decent work, employee retention, precarious employment, South African financial services sector

## Abstract

**Introduction:**

The South African financial services sector currently grapples with employee turnover, as increasingly flexible work arrangements have triggered more casual labor due to indecent work practices including poor compensation and a lack of job security which compromise employee retention. This research aimed to understand how employee retention can be improved in the South African financial services sector by promoting decent work.

**Methods:**

A qualitative approach was adopted to understand the research phenomena, specifically using a case study research strategy. A purposive sampling technique was utilized to select the 17 employees within the South African financial services institution who were interviewed through semi-structured interviews.

**Results:**

Following the deductive coding techniques that were used to conduct the thematic data analysis, the current state of decent work in the South African financial services institution was characterized by the work policies and working conditions experienced by employees, compensation and benefits, career development and growth, as well as organizational culture and management practices. Meanwhile, the mechanisms that were identified as enhancing employee retention by promoting decent work included competitive pay, rewards and recognition, professional development, job security and employment stability, as well as the institutionalization of flexible work arrangements.

**Discussion:**

This research offers empirical insights on how South African financial institutions can improve their employee retention by promoting practices that encourage decent work. Practical recommendations proffered by the study include implementing transparent compensation and recognition outcomes, promoting equitable access to employee development opportunities, officializing flexible work structures, and fortifying employment stability and job security.

## 1 Introduction

Financial services institutions globally continue to grapple with employee retention challenges, especially amid rapid technological advancement and innovation, the growing war for specialized talent, as well as employees' increasing pursuit of meaningful work that supports their wellbeing (Al-Azzam and Obeidat, 2025; El Mountasser and Sahraoui, 2025). Within such global contexts, decent work continues to grow in importance as a crucial organizational resource due to its notable influence on employee wellbeing, enhanced commitment, and amplified organizational sustainability (Musevenzo et al., 2024; Rantanen et al., 2020). Also related to decent work, studies internationally demonstrate that when employees experience equitable compensation, safe and supportive working environments, realistic workloads, access to advancement opportunities, and a sense of dignity in their vocation, they are more likely to remain loyal to their organizations and make positive contributions to their organization's performance in the long-run (El Mountasser and Sahraoui, 2025; Griep et al., 2025). Consequently, organizations that uphold decent work practices are more likely to experience enhanced employee retention, successfully safeguard their institutional competitiveness, and strengthen their resilience and sustainability in the volatile labor markets within which they operate (Mabaso and Booi, 2024).

The issue of employee retention in the financial services is clearly a global phenomenon (Al-Azzam and Obeidat, 2025; Sija, 2021), and the South African financial services sector—including banking, insurance, and pension funds—which plays a pivotal role in supporting the country's Gross Domestic Product, is no exception (Financial Sector Conduct Authority, 2022; Nesindande et al., 2024; Ngobeni et al., 2022). Although the sector is generally regarded as robust and resilient, even amid recent global market instability (Vilakazi et al., 2026), its sustainability is increasingly threatened by weaknesses in talent management. Van der Merwe and Malan (2020) found that turnover intentions among financial services employees in South Africa have been increasingly high, demonstrating the urgent need for mechanisms to curb this trend. Additionally, the Crowe (2023) Bank Compensation and Benefits Survey revealed that the banking industry has been particularly affected by difficulties in retaining younger talent because of unattractive compensation, unsatisfactory benefits, and limited career progression opportunities. Turnover intentions linked to the pursuit of better work–life balance also appear to be more prevalent among women, highlighting the need for retention strategies that respond to the diverse needs of employees (Villacé-Molinero et al., 2024). The implementation of such mechanisms is especially important because successful employee retention strengthens organizational performance and, in turn, enhances the competitive advantage of financial services institutions (Sepahvand and Khodashahri, 2021).

Employee turnover also carries consequences that extend far beyond recruitment costs. Zulkarnain et al. (2025) allude to additional challenges, including the costs of training and developing new employees, loss of income, and declining morale among staff who remain in the organization. Hughes et al. (2021) also highlight that employees who stay after a season of increased employee turnover within the organization often become demoralized and overloaded with added responsibilities to make up for those departed, which often reduces productivity and undermines service quality. Such challenges emphasize the importance of retention strategies that not only support organizational performance, but also promote decent work as a strong basis for employee wellbeing and institutional sustainability.

Meanwhile, the International Labor Organization (ILO) introduced the Decent Work Framework in efforts to minimize the decline of employment conditions by promoting employment that adheres to acceptable standards while safeguarding employees who are at risk of exploitation (International Labour Organisation's, 1999). This framework is embedded within the United Nations 2030 Sustainable Development Goals and offers guidelines for upholding fair labor practices, while safeguarding employees' human rights within the workplace; thus, noncompliant organizations may be sanctioned (United Nations, 2015). The Decent Work Framework primarily emphasizes equity, freedom, human dignity, security and values; thus, underscoring the standards for employment equity in modern day organizations.

Although various studies worldwide address how indecent work directly affects employee retention (Chada et al., 2022; Chikove and Hofisi, 2025; Kaan Namal et al., 2024; Wang et al., 2019; Zehri et al., 2024), few such studies have been conducted in South Africa, particularly in the financial services sector (Vilakazi et al., 2026). For example, several South African studies have explored how aspects like organizational culture and equitable compensation relate to employee retention (Maloba and Pillay-Naidoo, 2022; Maphanga and Olivier, 2025; Potgieter and Snyman, 2018; Van der Merwe and Malan, 2020), but not explicitly in accordance with the ILO's decent work framework. Meanwhile, Saurombe and Barkhuizen (2022) examined the association between happiness, meaningfulness and the intention to quit, although decent work is also not explicitly examined in the study. Another study by Hammond and Coetzee (2022) highlighted retention enablers, including equitable pay, favorable working conditions, and ongoing opportunities for career advancement; however, these retention enablers are not explicitly linked to decent work in the study. Additionally, a noteworthy portion of decent work studies seems to be more prevalent in sectors such as hospitality, where precarious work has been a longstanding challenge (Deery and Jago, 2015; Gupta, 2019), while similar research in the financial services sector remains limited thus far. Hence, this research contributes to addressing these gaps by extending the authors' existing work, amid growing interest in research on decent work, as such studies remain limited within the South African financial services sector (Van der Merwe and Malan, 2020; Vilakazi et al., 2026).

### 1.1 Research purpose and objectives

This research aimed to understand how employee retention can be improved in the South African financial services institution by promoting decent work. The research was based on one South African financial services institution where deficiencies in decent work practices compromised employee retention.

The specific objectives were to:

- establish the current state of decent work in the financial services institution, and
- explore the mechanisms to enhance decent work while improving employee retention in the financial services institution.

### 1.2 Research problem

South Africa's employment landscape has been substantially altered by technological advancement and globalization (Naidu et al., 2026; Nesindande et al., 2025). Particularly due to these alterations, various sectors currently grapple with upholding acceptable labor standards, thus adversely affecting the conditions workers are subjected to while threatening their right to dignity through decent work, and the financial services sector is no exception (Vilakazi et al., 2026). Meanwhile, the noteworthy lack of decent work in the South African financial services sector due to fast-paced technological advancement, which gives rise to worker displacement and redundancy, job insecurity, as well as precarious and casual employment, remains problematic, especially where companies are striving to improve their employee retention (Dabai and Novel, 2022; McWha-Hermann et al., 2025). Such challenges, therefore, substantiate the need for further scientific research to be conducted toward enhancing employee retention through decent work in the South African financial services sector.

## 2 Literature review

### 2.1 Theoretical foundation

The present research adopted the foundational principles of the decent work framework which focuses on key constructs including career advancement, equitable remuneration and job security, while simultaneously leveraging the psychological contract theory to underpin the positive effect a perceived decent work experience among employees is shown to have on employee retention. This theoretical framework adopted in the research is subsequently outlined.

#### 2.1.1 The decent work framework

The ILO's decent work framework underpins the understanding of what constitutes acceptable employment standards in contemporary workplaces. Established in 1999, the framework confronts the damaging effects of unfavorable working conditions while encouraging employment characterized by decent standards. The framework's embedding into the 2030 Sustainable Development Goals agenda, particularly SDG 8, demonstrates its worldwide importance in advancing fair labor practices and safeguarding workplace human rights (International Labour Organisation's, 1999; United Nations, 2015).

Decent work entails various facets such as promoting equitable compensation, improving the conditions of work, advancing access to health and safety facilities and preserving people's rights (Blustein et al., 2019). Furthermore, decent work constitutes personal employee aspirations to perform their work well, as well as advance in their career as a consequence of such good performance (Huang et al., 2022). Duffy et al. (2019, 2021) and Navajas-Romero et al. (2022) suggest that workplaces that uphold equitable policies and procedures, work–life balance, as well as occupational health and safety (key factors associated with decent work) stand a better chance at successfully retaining their employees.

Meanwhile, in workplaces where decent work is deficient, this gives rise to precarious employment characterized by workplace instability, job insecurity, and market unpredictability (McWha-Hermann et al., 2025), particularly in the financial services sector (Vilakazi et al., 2026). Shin et al. (2023) further highlight how employees experience job insecurity, professional instability and financial strain when their employment experience is haracterized as precarious, as conventional employment benefits such as paid leave, medical insurance and retirement packages tend not to be accessible to precariously employed workers. These workers are therefore compelled to juggle more than one job or work overtime to make ends meet. Thus, the decent work framework employed in this study promotes the understanding of how the satisfactory employment standards of employees within the South African financial services institution can be upheld by offering them freedom, security, equity and human dignity (decent work principles).

#### 2.1.2 The psychological contract theory

The psychological contract theory pertains to the unspoken, implicit expectations, convictions, and responsibilities that exist between workers and employers (Nnaji-Ihedinmah et al., 2021). In practical terms, this means that employees continuously evaluate whether the organization has fulfilled its perceived promises, while employers likewise expect employees to reciprocate through performance, loyalty, and commitment. It is generally accepted that the concept of the psychological contract was first established by Chris Argyris in 1960, and later substantially refined in 1989 by Denise Rousseau (Rajalakshmi and Naresh, 2018; Topa et al., 2022). The concept includes the shared understanding of commitments, responsibilities, and the perceived equity of the employment relationship (Ngobeni et al., 2022). When employees perceive that these obligations are being honored, they are more likely to experience trust, satisfaction, and a sense of belonging; whereas when they perceive breaches or imbalance, these positive attitudes may weaken (Bai et al., 2025; Mabaso and Booi, 2024). Several studies have shown that psychological contract, in turn, influences factors such as commitment, work engagement, job satisfaction, and employee retention (Nnaji-Ihedinmah et al., 2021; Okolie and Memeh, 2022). Decent work practices play a crucial role in shaping employees' perceptions of the psychological contract, leading to increased satisfaction and employee trust (Vilakazi et al., 2026). This is because decent work signals that the organization is meeting its implicit obligations to provide fairness, dignity, security, and meaningful employment conditions, thereby strengthening employees' belief that the organization is acting in good faith (Benavides et al., 2022; Mabaso and Booi, 2024).

Meanwhile, advancements in technology and unstable work arrangements are leading to a growing number of job displacements, leaving employees susceptible to disgruntlement concerning their psychological contract with their employer. Nnaji-Ihedinmah et al. (2021) mention that the psychological contract has noteworthy influence on employee loyalty in the financial services sector. Okolie and Memeh (2022) additionally aver that the psychological contract can be viewed as an integral component of an organization's strategy to retain highly skilled people to achieve corporate objectives and to enhance organizational effectiveness. Thus, the psychological contract theory enhances the understanding of how perceptions of a decent work experience among employees in the South African financial services institution affect employee retention, because decent work is expected to shape employees' perceptions of whether the organization has fulfilled its obligations (Bai et al., 2025), and these perceptions are then likely to influence job satisfaction, commitment, and the intention to remain with the organization.

### 2.2 Decent work in the financial services sector

Several studies have found that a general decline in the quality of work can be observed in the predominance of informal employment and the increasing flexibilization of jobs over the years (Jaiswal, 2019; Olubiyi, 2023; Ludwig and Webster, 2020). Jaiswal's (2019) study found that despite being perceived as a formal and regulated industry, the banking sector failed to meet the necessary standards for decent work. The study further illustrated that employees in the banking sector who were informal workers did not experience decent working conditions compared to their permanent counterparts. Although the number of studies on decent work has increased over time, a lack of understanding of the phenomenon persists in the financial services sector (Vilakazi et al., 2026). Hence, it is increasingly important to comprehend the significance of decent employment in the financial services sector (Jaiswal, 2019).

Recent sectoral employment data reveal the volatility that faces the financial services sector. Statistics South Africa (Stats SA)'s. (2024, 2025) quarterly reports show significant fluctuations in finance sector employment, with gains of 237,000 jobs in Q3 of 2023, followed by an additional 128,000 jobs in Q4 of 2023. However, there were losses of 189,000 in Q3 of 2024, followed by a recovery with gains of 60,000 in Q1 of 2025. This volatility raises concerns about employment security in the sector and underscores the importance of implementing decent work as a retention strategy. Meanwhile, a study by Wang and Cheung (2024) suggests that decent work indicators can play a crucial role in retaining employees. Studies have suggested that ensuring decent working conditions may have a significant impact on employee satisfaction, wellbeing, engagement, and retention (Wang and Chen, 2024; Ludwig and Webster, 2020). Sija (2021) similarly found that providing decent work conditions has been associated with improved levels of employee satisfaction. Thus, understanding the mechanisms that promote employee perceptions of decent work is beneficial for enhancing retention in the South African financial services sector.

### 2.3 Employee retention in the financial services sector

Employee retention represents a critical strategic imperative for financial services organizations operating in today's talent-driven economy (Ernest and Young, 2023). Maintaining a competitive advantage requires retaining competent workers whose expertise and core capabilities prove essential for success and sustained development (Muthuku, 2020). The financial services sector, in particular, emphasizes retention due to the significant direct and indirect costs associated with attrition (Roodt, 2018).

Multiple factors influence employee satisfaction and retention in the financial services sector. Bhardwaj et al. (2020) identify working hours, conditions, pay, benefits, job design, career advancement opportunities, and demographic characteristics (such as age, gender, and education level), human resources department quality, supervision, and stress as key determinants. Hammond and Coetzee (2022) emphasize that perceived retention enablers include conducive work conditions, fair compensation, and continuous career development opportunities. The strong connection between compensation, benefits, and job satisfaction has a significant impact on retention decisions within the industry (Sija, 2021).

Job satisfaction correlates with increased productivity, organizational loyalty, enhanced performance, and reduced turnover (Bhardwaj et al., 2020). Furthermore, satisfied employees provide superior customer service, creating positive organizational outcomes. Conversely, job dissatisfaction and unfavorable working conditions contribute significantly to attrition (Maloba and Pillay-Naidoo, 2022). Wang and Chen (2024) found that organizations promoting decent working conditions successfully decrease high employee turnover rates. Accordingly, organizations must adopt innovative strategies to retain talent through improving work conditions, promoting supervisor support, and funding employee development opportunities (Wanyama et al., 2025).

### 2.4 Best practices to ensure employee retention through decent work in the financial services sector

To ensure that the financial services industry follows principles of decent work, a variety of initiatives that address different areas of the work environment must be established. Integrating decent work principles into employee retention initiatives within the financial services sector is essential to cultivate a motivated, engaged, and loyal staff (Kabir et al., 2023). The interventions need to be aligned to the four pillars of decent work, namely employees, social dialogue, social security and workers' rights. Jaiswal's (2019) study reported that employers in the financial services business can retain personnel by ensuring competitive and equitable compensation based on responsibilities, experience, and market norms. Transparent remuneration frameworks foster confidence and diminish employee turnover. Studies have shown that job security and career development can have a positive impact on the retention of staff in the financial services sector (Houssein et al., 2020; Kabir et al., 2023). Organizations that provide long-term or permanent contracts mitigate job uncertainty (Jaiswal, 2019). Employees are more inclined to exhibit loyalty to an organization that offers stability and opportunity for career advancement. According to Wang and Chen (2024), in career development, greater emphasis should be placed on employee training and development, facilitating their career planning to achieve personal growth and self-worth. The financial services sector could also benefit from implementing continuous learning opportunities.

The financial services sector should develop and communicate clear career progression opportunities. Employers should consistently evaluate and revise career development programs to ensure alignment with employees' ambitions and competencies (Houssein et al., 2020). Studies in the financial services sector have also affirmed that employee retention is significantly influenced by career advancement opportunities, indicating that organizations offering such prospects are less likely to experience unmanageable turnover (Houssein et al., 2020; Kabir et al., 2023). The study by Saurombe and Barkhuizen (2022) suggested that supportive leadership, benefits and incentives, and training initiatives may mitigate turnover. Employers can also benefit by implementing flexible work arrangements, specifically in a fast-paced environment such as the financial services sector (Nesindande et al., 2025). Employers should establish flexible working hours, remote work alternatives, and reduced workweeks to assist employees to reconcile personal and professional obligations. Adopting these best practices would foster a work environment that not only attracts talent, but also promotes long-term employee commitment (Saurombe et al., 2022). By adhering to the principles of decent work, financial institutions may improve job satisfaction, decrease turnover, and cultivate a more resilient and engaged workforce.

While flexible work arrangements offer numerous benefits for employee retention and work–life balance, remote work is not universally preferred. BambooHR's (2024) Return to Office survey found that some employees prefer office-based work owing to home distractions (11%), inadequate equipment (13%), and family disruptions, with 74% reporting better performance in the office. Sutarto et al. (2022) identified key remote work challenges, including a lack of technical equipment, overlapping work-personal boundaries, and unstable internet connections, issues particularly relevant for South Africa's context, where load shedding and inconsistent connectivity impair remote work viability. Kong et al. (2022) note that workplace preferences are highly individualized. Therefore, financial services organizations should implement flexible work policies that offer employees choice rather than mandates, recognizing that different employees thrive in different environments.

## 3 Methodology

The following subsections delineate the philosophy of the research, the specific technique used to develop the research findings (deductive reasoning), the various methodological assumptions, the research strategy, data collection methods, ethical considerations, strategies employed to ensure trustworthiness of the data, as well as the data analysis techniques used.

### 3.1 Research philosophy

This study adopted a phenomenological approach to examine participants' lived experiences of decent work and retention. An interpretivist ontological perspective facilitated insight into participants' subjective realities, organization that these experiences are socially constructed. From an epistemological standpoint, the phenomenological approach enabled the researcher to capture the core essence of participants' experiences of decent work and retention, allowing for an in-depth exploration of how meaning is constructed within their organizational context.

### 3.2 Research approach

A qualitative research approach was deemed most suitable for this study, as its purpose was to describe conditions and phenomena (Creswell and Poth, 2018). This approach facilitated a comprehensive and nuanced exploration of participants' perceptions and experiences of decent work, as well as the complex factors shaping their decisions to remain within their organization based on these perceptions and experiences.

### 3.3 Research strategy

This study employed a case study strategy, informed by the authors' observation of growing retention challenges linked to decent work factors within the South African financial services sector, and more specifically, within the institution under exploration. As noted by Annamalah et al. (2025) and Takahashi A and Araujo (2020), case study research is widely valued for its capacity to facilitate in-depth exploration and contextualized understanding of complex, real-world phenomena, particularly in business and social science contexts. It also supports the development of practical, tailored solutions to specific organizational challenges, while providing a strong basis for further investigation in comparable or varied settings (Takahashi A and Araujo, 2020). In this study, this was realized through the generation of context-specific recommendations for the selected institution, which may be further examined and tested by researchers in similar or alternative environments in relation to the research topic.

### 3.4 Research participants and sampling

The study population consisted of 17 employees from a South African financial services organization, selected using purposive nonprobability sampling. This approach facilitated the identification of information-rich participants, offering meaningful insights into the phenomenon that was explored (Nayak and Singh, 2021). Participants were drawn from three departments—contact center (5), operations (7), and support (5)—thereby ensuring a range of perspectives across different organizational functions.

The sample included 11 permanent and six temporary employees, intentionally chosen to reflect experiences across varied employment categories. Participants' length of service ranged from less than 1 year to more than 20 years, while overall industry experience spanned between 5 and 34 years. This diversity enabled the inclusion of both early-career and highly experienced employees, providing insight into how perceptions of decent work and its influence on retention may vary according to tenure within the financial services sector.

Participants' educational backgrounds were diverse, ranging from matric certificates to undergraduate degrees, postgraduate qualifications, and professional certifications such as RE1, RE5, and accreditation from the South African Institute of Chartered Accountants (SAICA). As defined by the South African Qualifications Authority (2025), matric certificates are awarded upon completion of secondary schooling; basic degrees refer to undergraduate qualifications; advanced degrees include honors, postgraduate diplomas, and doctoral-level studies; and professional accreditations are obtained through recognized bodies such as SAICA. A notable number of participants were actively engaged in further studies, reflecting a strong commitment to personal development. In terms of work arrangements, most participants operated within hybrid models that combined office-based and remote work, while others remained in traditional office settings or occupied specialized field-based roles.

### 3.5 Data collection

Semi-structured interviews were conducted with each participant, lasting approximately 60 min on average. This duration allowed for an in-depth exploration of participants' experiences while remaining mindful of their time constraints, and is considered appropriate for qualitative research (Ugwu et al., 2021). The interview questions were designed in alignment with the study's objectives and informed by the literature review, which highlighted factors such as fair remuneration, job security, opportunities for career progression, and work–life balance as central to both decent work and employee retention (Demiral et al., 2022; Sija, 2021; Wang et al., 2019). The semi-structured format provided the flexibility to explore key themes while maintaining focus on the research aims, and allowed for probing of responses while ensuring consistency across interviews. Sample questions included: What do you consider to be the key elements of “decent work”? Which aspects of a job make you feel valued as an employee? How would you describe working conditions in the financial services sector based on your experience? How do you perceive fairness in terms of compensation and promotion in your current role? What factors would influence your decision to remain with your current employer? What strategies would you recommend to enhance decent work and improve employee retention within your organization?

Most interviews were conducted in prebooked boardrooms at the organization's premises to ensure privacy. However, some interviews were held virtually via Microsoft Teams, depending on participants' preferences and availability. All interviews were audio-recorded with participants' consent, obtained both through signed informed consent forms and verbal confirmation at the start of each session. Interviews were transcribed verbatim to preserve the authenticity of participants' responses. In addition, field notes were used to document nonverbal cues, emotional expressions, and contextual elements that may have influenced responses. To ensure confidentiality, pseudonyms were assigned to all participants, thereby fostering a safe environment for open and honest discussion of potentially sensitive workplace issues.

### 3.6 Ethical considerations

Following approval of the research proposal, the authors sought and obtained ethical clearance from the University of Johannesburg (UJ) Ethics Committee prior to commencing the study. Permission to conduct the research was also requested from the HR Director of the selected organization within the South African financial services sector. The authors assured both the organization and its employees that anonymity would be maintained in accordance with the Protection of Personal Information (POPI) Act of South Africa.

Participation in the study was entirely voluntary, and participants were informed of their right to withdraw at any stage of the research process without consequence. Before taking part, all participants were provided with detailed information regarding the purpose and requirements of the study and were required to sign informed consent forms to confirm their voluntary participation and ensure confidentiality. The researchers ensured that the potential benefits of the study outweighed any possible risks, and that both advantages and disadvantages were fairly distributed among participants.

All data collected was used strictly for the purposes of this study. To protect participants' dignity and prevent any harm, the data was securely stored in a locked safe at the researchers' residence and will be retained for a minimum of 5 years, in accordance with UJ's research data management and storage guidelines.

### 3.7 Data trustworthiness

Various measures were employed to ensure methodological rigor in this research, particularly according to Lincoln and Guba (1985) proposed trustworthiness criteria encompassing credibility, transferability, dependability, and confirmability. The authors endeavored to maintain objectivity throughout the study by avoiding leading or biased questions and refraining from imposing their own views on participants during the interviews (Creswell and Poth, 2018). To enhance the study's credibility, the researchers provided a detailed account of the research methodology as well as participants' backgrounds, and ensured the study was conducted rigorously (Stahl and King, 2020). Credibility was further enhanced by selecting participants who possessed direct knowledge and experience of the phenomenon under investigation, thereby enabling the collection of rich and relevant data. All interview-related materials, including transcripts and findings, were securely stored to allow for future reference and potential auditing. This contributed to the study's confirmability by ensuring that the findings could be traced back to the original data and that an audit trail was maintained throughout the research process (Lincoln and Guba, 1985).

The researchers also employed reflexivity across the research process, engaging in attentive listening to fully understand participants' experiences without forming preconceived judgements (Lincoln and Guba, 1985). The use of reflexivity further strengthened confirmability by minimizing the potential influence of researcher bias on the interpretation of the findings. Transferability was enhanced through the provision of detailed and transparent descriptions of the research context, participants, and procedures, enabling readers to assess the applicability of the findings to similar settings (Lincoln and Guba, 1985). Dependability was supported through the systematic documentation of the research design, data collection procedures, and data analysis process, thereby ensuring consistency and transparency throughout the study and enabling the research process to be reviewed by other scholars (Lincoln and Guba, 1985).

### 3.8 Data analysis

Data analysis was conducted in accordance with Braun and Clarke's (2021) six-phase thematic analysis process. This involved firstly familiarizing oneself with the data through repeated readings, secondly, systematically generating initial codes across the dataset, thirdly, identifying potential themes by grouping related codes into subthemes, fourthly, reviewing these subthemes in relation to both the coded extracts and the full dataset, fifthly, refining and naming the subthemes to reflect their core meaning, and sixthly, producing the report supported by illustrative extracts. A predominantly deductive approach was employed, whereby existing literature informed the development of the empirical findings. In this context, deductive reasoning entails a theory-driven interpretation of data, using established knowledge to guide analysis, in contrast to inductive approaches that generate findings purely from the data itself (Braun and Clarke, 2021).

All authors participated in the analysis, each undertaking independent coding processes documented in separate hardcopy journals. These individual analyses were later compared, discussed, and integrated through consensus to arrive at the final thematic structure. While the themes and subthemes initially identified by each author were largely aligned, agreement was primarily required regarding their wording and presentation.

Manual analysis was intentionally selected over software-assisted techniques to facilitate deeper engagement with the data. This approach allowed the researchers to remain closely connected to participants' perspectives and to capture nuanced meanings and contextual subtleties that automated tools might overlook (Korstjens and Moser, 2018). Although more time-intensive, manual analysis enhanced interpretive depth, which is critical in qualitative research, and ensured an authentic representation of participants' experiences through direct interaction with the data (Busetto et al., 2020).

## 4 Findings

The findings are presented in two parts: first, an overview of the participants' biographical information, and second, an analysis aligned with the study's two primary objectives, namely, to establish the current state of decent work in the financial services institution, and to explore the mechanisms to enhance decent work while improving employee retention in the financial services institution. Each subtheme under the two main themes is substantiated with participant narratives that shed light on the complex realities of working within this organizational context.

Specifically, the subthemes presented under theme one include: work policies and working conditions; compensation and benefits; career development and growth; as well as organizational culture and management practices. Overall, these subthemes demonstrate how the current state of decent work in the South African financial services institution is substantially influenced by work policies and arrangements, remuneration (both monetary and non-monetary), career growth opportunities, and organizational culture and management behaviors that are often either favorable or unfavorable, particularly concerning decent work and its ultimate influence on employee retention. Meanwhile, the subthemes presented under theme two include professional development, job security and employment stability, as well as the institutionalization of flexible work arrangements, specifically reflecting the key mechanisms which the financial services institution can adopt to enhance their employees' perceptions and experience of decent work, which in turn would enhance the retention of these employees.

### 4.1 Biographical data of the research participants

With deliberate measures taken to ensure that participants could not be easily identified and that their privacy was protected in line with the POPI Act of South Africa, biographical data were collected to enrich and contextualize the findings. This information included participants' gender, job roles, years of experience within the financial services sector, employment status, and working arrangements. Table 1 below presents an overview of the participants' profiles in this respect.

**Table 1:** Participants biographical data.

Pseudonym	Gender	Roles/responsibilities	Tenure in the financial services	Employment type	Employment model
P1	Female	Contact center – client services	1 year	Permanent	Hybrid
P2	Female	Contact center – client services	4 years	Permanent	Hybrid
P3	Female	Contact center – client services	5 years	Permanent	Hybrid
P4	Male	Contact center – client services	23 years	Permanent	Hybrid
P5	Female	Operations – admin	17 years	Permanent	Hybrid
P6	Male	Client services – branch admin	24 years	Permanent	Traditional, office-based
P7	Male	Support – finance	12 years	Permanent	Hybrid
P8	Female	Operations – broker support	13 years	Permanent	High mobility hybrid
P9	Female	Operations – sales	34 years	Permanent	Hybrid
P10	Male	Operations – sales	13 years	Permanent	High mobility hybrid
P11	Female	Support – admin	1 year 5 months	Temporary	Hybrid
P12	Male	Support – legal	5 years	Temporary	Traditional, office-based
P13	Female	Support – HR	1 year 7 months	Temporary	Hybrid
P14	Female	Support – admin	12 years	Temporary	Traditional, office-based
P15	Female	Support – admin	15 years	Temporary	Traditional, office-based
P16	Female	Support — admin	11 years	Permanent	Traditional, office-based
P17	Female	Support – admin	8 years	Temporary	Traditional, office-based

Authors' own compilation.

### 4.2 Theme 1: the current state of decent work in the South African financial services institution

The first research objective was to understand employees' current lived experiences of decent work in the financial services institution. This was achieved by exploring how the various dimensions of decent work translate into tangible workplace realities that shape employees' daily employment experiences. Moving beyond perceptions and ideals, this analysis focuses on the actual provision of decent work conditions and how employees experienced these conditions in their concrete work situations. Based on what participants were asked about the current state of decent work in the financial services institution, the following subthemes were formed: work policies and working conditions, compensation and benefits, career development and growth, organizational culture and management practices.

#### 4.2.1 Subtheme 1: work policies and working conditions

This subtheme highlights the diverse employee experiences and perceptions of work policies and working conditions in the financial services institution, revealing how the physical, psychological, and organizational environment influences their understanding and perception of decent work. This subtheme revealed how workplace policies, organizational initiatives, and various working conditions affect employees' experiences of decent work, by highlighting specific policies that either enhance or limit comprehensive decent work provision within the organization.

Flexible work arrangements have become one of the most esteemed organizational policies that enhance the provision of decent work, according to some of the participants. For example, P3 described the transformative impact of remote work policies on their work experience:

“*Work from home is perfect for me. That 5,000 rand I would have spent on transport I can use to pay my sister's residence fees and give her spending money. There is a level of trust here—most companies would not trust you to work from home and only come in two days*.”

Meanwhile, some participants were positive about the wellness policies instituted through employee wellness programs in their organization, which they considered to have a noteworthy contribution to decent work. For instance, P16 declared:

“*We have the company's wellness program, which is very nice. When you get stressed, you can get free counselling. That is an excellent program the company has for us*.”

P11 further expressed:

“*I would say the wellness day; I think that is a good one because it shows that they are very much interested in knowing about our health and wellbeing as employees*.”

However, several participants did not initially mention the wellness programs available within their institution, and upon the researchers' probing, the participants revealed that there was limited awareness of these programs or poor communication about these initiatives.

Some participants highlighted their experience of fair HR policies, which they recognized as contributing to decent work environments. P7 particularly noted the importance of clear, consistently applied policies:

“*Having proper HR policies* [is important]. *If you do certain things, there will be disciplinary hearings, so you tend to make sure you do not break those rules. This contributes to a good working environment*.”

However, participants generally displayed unfamiliarity with workplace policies that promote decent work within the financial services institution, as demonstrated by P12′s following remark:

“*I do not think there is anything* [specifically referring to workplace policies and initiatives that articulate decent work].”

The above discourse demonstrates how formal policies and initiatives such as flexible work arrangements, extensive employee wellness programs, and sound human resources policies are essential facets of decent work in organizations. However, the findings revealed deficiencies in the outright articulation of decent work, as numerous employees were unaware of decent work policies specifically and intentionally instituted by their organization to enhance their overall work experience.

Regarding the current working conditions experienced by employees in the financial services institution in relation to decent work, some participants highlighted working conditions that satisfy the standards of decent work, while others noted conditions that fall short in upholding basic decent work principles. Exploitative practices in financial services organizations emerged as a concern, with one participant describing the sector as quite exploitative. Various participants specifically reported challenging working conditions, including excessive workload, management issues, competitive pressure, industry-specific challenges, and demanding sector characteristics.

For example, P3 stated:

“*The financial services sector is very exploitative… it is like in this industry, sometimes you end up being lucky if you end up in a proper environment; basically, it works with luck. I went for an interview, and they were willing to offer me 2000 rand as a basic salary. In this current economy, can you offer someone 2000 rand*?”

P7 further remarked about excessive workload and pressure becoming prevalent factors that affect employee wellbeing:

“*I think, in general, there is too much pressure… You find yourself doing more work than two or three people could do, but you are doing it all alone simply because of the demand. So, now you are neglecting your personal time, working overtime, and weekends*.”

P6 further emphasized the competitive nature of the sector and stated:

“*Competitive*, [the financial services sector is] *very competitive. Definitely, there is much competition when it comes to sales; so, internally, between different groups, there is competition as well as externally with different companies competing to become the best financial services provider*.”

Notwithstanding the above-outlined shortcomings in working conditions, some participants acknowledged positive aspects of physical working conditions, though they noted that management's behaviour ultimately substantially affected the overall work environment. P9 particularly stated:

“[physical] *Working conditions* [in my organization] *are good—you have good lighting, air conditioning, all the physical amenities. However, the challenging part is management. If somebody is shouting and screaming and walking around in a lousy mood, it is not going to be good working conditions; regardless of how great the office facilities are*.”

Meanwhile, P10 posited that organizational support helps to mitigate pressures in the sector:

“*The financial services sector requires long hours—it is what it is. It is fast-paced and high-pressure due to intense competition. However, there is also professional support given to make sure we do not burn out*.”

Based on this subtheme's overall discourse, participants' experiences of work policies and working conditions in the financial services institution vary widely, particularly when it comes to decent work, ranging from positive to negative. The diversity of the participants' responses suggests that decent work experiences are substantially affected by specific organizational nuances and personal experience, rather than being consistent across the institution.

#### 4.2.2 Subtheme 2: compensation and benefits

Another subtheme that emerged under the current state of decent work in the financial services institution, based on the lived experiences of the participants, is compensation and benefits. The focus of this subtheme includes fairness, transparency, and appropriateness of financial remuneration and related benefits. Various participants revealed significant variations in how different employee groups experience this aspect of decent work in their organizations, with particular emphasis on how employment status affects access to fair compensation and other benefits packages. The lack of transparency in compensation schemes was particularly noted as a critical obstacle to perceived fair compensation. In this regard, P7 stated:

“*It is difficult to answer because fairness comes when you know what others who are doing the same job, are paid. Unfortunately, salaries are confidential, so you will never know if you are being paid fairly or not*.”

Another participant (P16) further raised concerns about the market competitiveness of compensation:

“*Compensation-wise, I do not think it is fair. I do not think what they are paying us is good compared to market rates, although I do not know how much others are getting paid*”.

Some temporary employees reported experiencing unfair compensation practices, particularly regarding overtime payment and benefits. For example, P12 expressed the following frustration:

“*No, I feel I am not compensated fairly. The workload is heavy. We work very long hours every month; sometimes until 8 pm or 9 pm, and sometimes on weekends. We work for free—*[there is] *no overtime pay*.”

Changes in compensation policies further exacerbated concerns about fairness among temporary workers. P14 described a sudden compensation policy change in the institution:

“*Until last November, we were paid overtime. Then HR told us: ‘No, we will not pay overtime anymore.' Previously, we were receiving more due to overtime, but now it is just our basic salary. I feel it is not working for us*.”

Meanwhile, some participants indicated more favorable experiences regarding compensation fairness, especially within the sales department. P9 expressed the following contentment with the fairness of compensation within their department:

“*For me*, [what is important] *is fair compensation for what I do. In sales, if you do not put in extra effort, your compensation goes down. In my current environment, the compensation is fair*.”

P5 further affirmed their perceived fairness of remuneration in the financial services institution by saying:

“*According to us in the call center, it is fair because we all start with the same basic salary, and as you stay with the company, we grow according to the percentage given, and the percentage is equal for everyone*.”

The participant views garnered in relation to this subtheme indicate that fair compensation and comprehensive benefits packages are essential elements of decent work; however, their provision varies significantly across employee categories and departments within the financial services organization. Hence, the findings reveal systematic gaps in compensation, which harm decent work, especially for temporary workers who are denied other benefits packages such as overtime pay and clear compensation structures.

#### 4.2.3 Subtheme 3: career development and growth

This subtheme considered career development and growth opportunities in the financial services sector, focusing on employees' access to skills development, promotion prospects, and professional advancement activities. The findings demonstrate how organizations either support or hinder employees' professional growth ambitions, directly influencing the quality of their work experience and adherence to decent work norms. The responses from participants revealed considerable inequalities in accessing developmental opportunities, with a clear divide between the experiences of permanent and temporary employees.

The exclusion of temporary workers from developmental opportunities particularly emerged as a critical issue that affects their experience of decent work. For instance, P12 expressed frustration with the systematic exclusion from growth programs that are available for permanent employees:

“*They do have a study loan, but they said that temps do not qualify for it—it is only for permanent employees. I will remain where I am until I do not know when, and I am not earning enough money to enrol in university myself*.”

P17 had similar concerns regarding prospects for development, and remarked:

“*For us, temps, we do not benefit from the study assistance*.”

P14 noted that it is helpful when management encourages temporary employees to further their studies in order to improve their prospects of career advancement and promotion when opportunities arise within the company:

“*Our manager has been encouraging us to get RE5 qualifications so we can move forward in this company. She is encouraging us to better ourselves so we can progress in the company instead of just telling us that when our contract ends; we will have to find another job*.”

Thus, while the financial services organization provides comprehensive development programs, these are primarily accessible to permanent employees. P2 specifically highlighted the range of development opportunities available to permanent staff:

“*The company offers programs for learnerships, and they allow you to do your NQF level 4 and NQF level 5. The company also offers bursaries for people to apply for their studies to be funded*.”

As a permanent employee, P3 also appreciated the soft skills training that the organization provided:

“*The soft skills training was essential for us to be self-aware and ensure we take care of ourselves so we can show empathy to clients. The course helped with email etiquette—now you know when you write, it needs to be professional*.”

P13 further described a meaningful development experience that was made available to her as a permanent employee:

“*Career development, I think, when I joined the training, for example, for soft skills, that was empowering because it challenged the inner person; you can see yourself and motivate and develop yourself*.”

Meanwhile, some participants were unaware of the available developmental opportunities. For instance, P7 admitted:

“*I am not aware of any programs or initiatives in the organization; I will be honest*.”

Similarly, P15 remarked:

“*I do not know, okay, let me say, I think every month we get these assignments. So, I guess I learn from that*.”

Summarily, career development and growth opportunities are essential elements of decent work, which enable employees to enhance their skills, advance in their professions, and achieve their professional goals. However, the findings revealed certain disparities in the lived experiences of various employee categories in the financial services institution, which have the potential to erode the principles of decent work. Put differently, the systematic exclusion of temporary workers from development opportunities creates a two-tiered system that contradicts the decent work principles of equality of opportunities. Furthermore, it appears that there are employees who lack awareness of the available developmental programs in the financial services institution, which suggests inadequate communication or limited availability of these programs.

#### 4.2.4 Subtheme 4: organizational culture and management practices

The subtheme organizational culture and management practices was also developed with respect to the current state of decent work in the financial services institution. The subtheme specifically considers the influence of organizational culture and management practices on employees' lived experiences of decent work. It focuses on the impact of leadership quality, workplace relationships, and organizational values in fostering or hindering decent work in the financial services institution. Managerial behaviour and organizational culture were considered to be essential factors of decent work, which influence employees' lived experiences in terms of dignity, respect, and fairness in their workplaces.

One participant (P4) described how supportive management practices can enhance their experience of decent work by alluding that positive management relationships can transform employment experience:

“*At first, it was job security—just having a regular income to provide for my family. However, now as time goes on, I am actually enjoying what I do and how my manager and leadership allow me to do it*.”

Some participants experienced organizational culture that aligns with decent work principles. For example, P16 expressed a strong emotional connection to her organization's culture:

“*I think the company feels like home. I like the current organization's culture—it feels like home. I like the way they serve their clients*.”

P13 further appreciated certain aspects of their organizational culture, particularly regarding formal communications, stating:

“*For me, the culture itself, the integrity, the transparency in communication regarding company events—they communicate prior to events. I do admire the relationship and professionalism of the company*.”

Furthermore, a sincere open-door policy and transparency in management practices were recognized as essential components of decent work, which was experienced by some employees in the financial services institution like P8, who noted:

“*There is an open-door policy established to voice concerns. You have confidentiality—whatever you say is treated confidentially. When you voice issues, you will not be subjected to discrimination or prejudice*.”

Notwithstanding the above favorable insights regarding the overall culture experienced by several employees within the financial services institution, some participants highlighted the need to revise some managerial practices that affect the lived experiences of employees when it comes to decent work. In this regard, P1 stated:

“*Maybe if they can change their management strategy and improve it. Furthermore, there is a need to provide a healthy working environment, where everyone feels free, and the support must not always be from the team, but management must also be there*.”

In essence, organizational culture and management practices play a significant role in fostering decent work in organizations, including those in the financial services sector, by enhancing human dignity, organizational justice, and the wellbeing of both professionals and individuals. However, the findings also revealed some deficiencies in management practices that undermine the provision of decent work, in which case there is clear room for improvement.

### 4.3 Theme 2: mechanisms to enhance decent work while improving employee retention in the financial services institution

The second research objective was to identify mechanisms for enhancing decent work while improving employee retention in the South African financial services institution. Based on what the participants were asked about possible mechanisms that could be adopted to enhance decent work and improve employee retention in the South African financial services institution, the following subthemes were formed: competitive pay, rewards and recognition; professional development, job security and employment stability, as well as the institutionalization of flexible work arrangements.

#### 4.3.1 Subtheme 1: competitive pay, rewards and recognition

This study found competitive pay, rewards and recognition as critical mechanisms for enhancing decent work and improving employee retention. The participants suggested that competitive compensation (including benefits) is a plausible means of enhancing decent work, which would in turn improve employee retention. For instance, P4 stated the following:

“*One mechanism that is important to me is competitive compensation; the company must pay me a market related salary for me to be happy and not leave*.”

P8 added:

“*The organization should pay a market-related salary, which is a vital thing for me; this is one thing that is important for me to be happy with the organization*.”

Meanwhile, P4 emphasized the important role competitive benefits play in retaining employees, over and above competitive pay:

“*I would suggest that getting competitive benefits would actually keep people happy in the organization. You will be in two minds before you leave a company that does provide extensive benefits rather than going to a company that pays a higher salary but does not have these benefits. So, providing competitive benefits, I would say, definitely matters to me*.”

Some participants further suggested specific financial benefits that would enhance decent work and improve their intention to remain with their *organization*, such as shares, employee discounts and financial assistance programs. In this regard, P1 suggested:

“*Maybe the company can offer employees shares when you exceed your performance. They can also maybe offer partnerships with other financial institutions, where we get a discount if you want to buy a car and a house*.”

P8 similarly posited:

“*I think a mechanism that could make people to be happy and stay with the perationali is to grant them some kind of subsidies... maybe give you discounts on certain home loans, your vehicle loans, things like that*.”

P2 additionally highlighted the motivational influence of regular performance-based incentives, which were perceived to serve as a noteworthy retention mechanism:

“*They must also offer the monthly incentives due to performance. That one I can say will make me happy and enjoy the environment*.”

Some participants suggested that temporary employees should be remunerated for overtime, whilst allowing them to access similar employee benefits as permanent employees in order to enhance their decent work experience and improve their retention, as seen in P15′s following response:

“[As a temporary employee] *Paid overtime would make me happy; it will make a big difference…. I would say medical aid, pension fund, those kinds of things would make me happy*.”

The above discourse demonstrates how participants considered competitive pay and benefits as powerful mechanisms to enhance decent work and employee retention in the financial services institution. Some of these suggested mechanisms include comprehensive benefit packages that may outweigh higher salaries elsewhere, performance-based incentives, employee shares tied to excellence, financial institution partnerships offering discounts on major purchases for employees, and market-related salaries that ensure fairness. Other participants proposed offering paid overtime, medical aid, and pension funds to temporary employees, similar to those provided to their permanent counterparts.

In the same breath, the findings revealed that rewards and recognition encourage positive perceptions of decent work, while enhancing employee retention. The participants consistently emphasized the motivational effect of recognition programs that include tangible rewards. In this regard, P2 stated:

“*I would be happy if they could offer trips as recognition—international or within Africa, like Cape Town—and vouchers based on performance*.”

Participants suggested that employee participation in recognition decisions could serve as a mechanism to enhance workplace democracy and dignity (which are core principles of decent work) while strengthening employee retention, as demonstrated by P3′s response:

“*Implementing peer nomination systems would give employees a voice in recognition processes, enhancing our sense of dignity and fairness at work, which would strengthen our commitment to stay with the organization*.”

P4 suggested that recognition programs should be accessible and achievable as a mechanism to enhance decent work and to improve retention:

“*Recognition programs should be attainable, which means that you should not set targets there for me that I need to walk on water before I can actually attain them; they should be reachable targets that everyone can attain*.”

In addition, the frequency of recognition emerged as a powerful mechanism to enhance decent work and to improve employee retention. P8 particularly advocated for regular recognition rather than once-off annual recognition:

“*They need to do this recognition of top performers in a department on a monthly basis….it makes people feel important so they need to do stuff like that more often as a mechanism*.”

Smaller and more frequent rewards were further suggested by P9 as an effective mechanism to enhance decent work and employee retention:

“*Smaller rewards help too. If you reward people weekly—like when they complete sales in the first 2 weeks instead of waiting until month-end—you could give household vouchers*.”

Nonmonetary appreciation through communication via email messages emerged as a powerful means to enhance the dignity of the working person, as well as decent work, which ultimately encourages employee retention. In relation to this, P14 stated:

“*One mechanism the company can use to retain their employees is to send emails of ‘thank you' to them—a small amount of such* [nonmonetary appreciation] *goes a long way. I want people to know I am working hard*.”

The findings thus revealed that financial services organizations should enhance their recognition systems, which are crucial mechanisms to enhance decent work and improve employee retention. Effective recognition systems could be operationalized through tangible rewards such as: trips and performance vouchers; participation in decision making by peers in the nomination systems to ensure fairness and transparency for accessible, achievable and realistic targets that relate to rewards and recognition; frequent monthly or regular recognition rather than once-off annual recognition; smaller and regular rewards that provide immediate satisfaction; and nonmonetary public appreciation through communications via email, which would enhance both the dignity of the working person as well as decent work, and ultimately encourage employee retention.

#### 4.3.2 Subtheme 2: professional development, job security, and employment stability

Professional development, job security, and employment stability were also found to be crucial in relation to mechanisms to enhance decent work and to improve employee retention. Importantly, some participants felt that their professional development directly influences their long-term employment outcomes, including the ability to secure and sustain permanent employment. In relation to how professional development enhances decent work while promoting employee retention, P5 stated:

“*The company should offer its employees more bursaries. Furthermore, there should be more room for people to go back to school; maybe tertiary, and get more degrees or qualifications, because that promotes career growth in a person'slife*.”

P4 similarly echoed that access to more qualification-culminating training courses is perceived as a plausible mechanism for enhancing decent work:

“*I prefer the organization to take me to training courses that offer formal qualifications I can add to my CV; so, these credentials are valuable*.”

Various temporary employees suggested that training and development opportunities could be used as mechanisms to support them to secure permanent employment. For instance, P15 stated:

“*They should offer bursaries so that we can learn and further our studies and then hopefully get permanent employment in the company… This is an important aspect of decent work and would make me want to stay…. If I have access to develop myself and grow within the company*.”

P13 additionally affirmed:

“*Providing study assistance to long-term temporary workers would create equality in development opportunities, a key decent work principle, while improving retention by showing that the organization values our growth and future contribution*.”

In as much as professional development was considered to enhance decent work and ultimately, employee retention, the upgrading and transferability of skills within the financial services sector was further emphasized as a mechanism to enhance decent work and promote employee retention due to the improved job security that such a mechanism provides. In this regard, P3 stated:

“*There is job security in the financial services sector, but it comes from your skills and experience, not your current workplace. If you have upskilled yourself, you can find work anywhere in the industry*.”

Meanwhile, P1 proposed the need to train employees in recent technological innovations such as AI, which enhances job security and employee retention:

“*The company can invest in AI training for us. This would enhance our job security by ensuring we remain relevant in the evolving workplace, which would significantly improve our desire to stay with the organization*.”

For temporary employees, the ability to secure permanent employment was considered a mechanism to enhance their experience of decent work in the financial services sector, as well as improve their possible retention. P15 specifically said:

“*For me, at this moment, being permanently employed would really change my life. It is an important thing for me*.”

P17 similarly reiterated:

“*I think if the company employed us permanently, that would improve my experience*.”

As per the above discourse, the findings revealed that professional development, job security and employment stability were considered as powerful mechanisms to enhance decent work and improve employee retention. This could be achieved through comprehensive bursary provision for employees to access tertiary education, thus enabling them to enhance their qualifications, as well as availing training courses that would result in formally recognized qualifications that employees can transfer to other roles or organizations. The findings further reveal that proactive training in recent technological innovations such as AI could serve as a mechanism to enhance employee job security. Moreover, temporary employees particularly considered training and development opportunities as plausible mechanisms for securing and sustaining permanent employment.

#### 4.3.3 Subtheme 3: institutionalization of flexible work arrangements

Another subtheme developed with respect to mechanisms to enhance decent work and improve employee retention is the need to institutionalize flexible work arrangements, which also prioritize worker wellbeing while creating effective retention mechanisms. The participants suggested that flexible work arrangements align with decent work principles, which directly enhance workers' wellbeing and serve as powerful retention mechanisms.

P2 specifically highlighted as follows, how remote work can enhance decent work and improve employee retention:

“*Maintaining the work-from-home policy would enhance decent work by ensuring reasonable working hours and reducing commute stress, which significantly improves my work–life balance and makes me committed to staying with the organization*.”

Correspondingly, P3 mentioned the impact of flexible work arrangements on decent work and employeeretention:

“*A hybrid working environment serves as a mechanism for decent work by providing flexibility that respects our personal lives while maintaining productivity. This balance between work and personal responsibilities strongly influences my decision to remain with the company*.”

In support of P3, P2 stated:

“*The company should maintain the 3-day remote, 2-day office arrangement, as this enhances decent work conditions by reducing fatigue and improving work–life balance, which our production data shows lead to both better performance and higher retention rates*.”

P4 particularly noted that the contact center's performance was higher during the weeks when employees came to the office less frequently. He stated:

“[Employees within] *The company can keep working from home for 3 days and 2 days in the office, because I have seen through my production reports that people in the contact center are more productive at home*.”

The findings suggest that work–life balance and flexible work arrangements are crucial mechanisms for enhancing decent work and improving employee retention. However, their effectiveness depends significantly on equitable availability and organizational support.

## 5 Discussion

This research aimed to understand how employee retention can be improved in the South African financial services institution by promoting decent work, while the specific research objectives were to establish the current state of decent work in the financial services institution, and to explore the mechanisms that could be adopted to enhance decent work while improving employee retention in the financial services institution. The discussion section of this paper follows the outline of the research objectives.

### 5.1 Objective 1: to establish the current state of decent work in the financial services institution

Exploring the current state of decent work revealed significant gaps between ideal conceptualizations and lived realities, with experiences varying dramatically based on employment status and organizational position.

#### 5.1.1 Work policies and working conditions: between exploitation and support

The current study's findings revealed significant gaps between policy provision and employee awareness and access to these policies. Flexible work arrangements emerged as a highly valued policy initiative. This supports Shekhar et al.'s (2025) and Wang and Cheung's (2024) observation that employees appreciate organizations that prioritize their overall wellbeing while allowing them to manage professional and personal commitments. Customized wellness programs were also appreciated; however, limited awareness of these programs among most participants suggests implementation failures that undermine decent work provision.

Development programs revealed stark disparities according to the participants. This aligns with Wang and Cheung's (2024) emphasis on employee training and development. However, the systematic exclusion of temporary workers contradicts fundamental decent work principles of equality, supporting Esieboma's (2020) observation about the limited prospects for temporary employees. Meanwhile, the limited awareness of decent work policies was indeed concerning. This suggests that even well-designed policies may fail to constitute decent work if employees are unaware of or unable to access them, highlighting the gap between policy intention and implementation that undermines decent work provision in the sector.

The findings revealed a spectrum of working conditions, which ranged from exploitative to supportive, though exploitation appeared to be more prevalent. This finding supports Ludwig and Webster (2020) observation about the highly adaptable labor market that emerged from the rapid expansion of precarious work. In addition, excessive workload emerged as a significant concern. While this finding aligns with Mosharrafa et al.'s (2025) finding that 93.45% of bank employees were experiencing burnout symptoms, with daily working hours and task variations identified as key contributing factors, it contrasts with Houssein et al.'s (2020) study, which found that some financial services employees remained engaged despite demanding conditions when other motivational factors were present. This divergence suggests that workload tolerance may vary based on individual circumstances and the presence of mitigating factors such as recognition, compensation, and career development opportunities.

However, some participants acknowledged favorable physical working conditions while identifying management as the critical variable. The finding suggests that decent work assessments that focus solely on objective conditions overlook crucial subjective dimensions shaped by interpersonal dynamics and leadership quality, which resonates with Huang et al.'s (2022) call for organizations to implement employee-oriented human resource management strategies to foster a favorable employee perspective of their work environment.

#### 5.1.2 Compensation and benefits: transparency and equity challenges

The absence of transparency in compensation emerged as a fundamental barrier to decent work. This opacity contravenes the International Labour Organisation (1999) principles of transparency and collective negotiation, thereby maintaining power imbalances that prevent workers from asserting their rights effectively. Meanwhile, temporary employees consistently reported unfair compensation practices. These experiences support Dabai and Novel (2022) assertion that some elements of decent work are denied to workers in casual employment relationships.

To reinforce our findings on exploitative practices and workload, we highlight that high work demands and precarious contracts are well-documented in finance. For example, Van der Merwe and Malan (2020) note that the “demanding and often challenging nature of work” in the South African financial services sector significantly increases employees' turnover intentions. International studies corroborate this: Dabai and Novel (2022) report that Nigerian banks increasingly rely on casual/contract labor and that these workers receive pay “not commensurate with the work done” while being deprived of benefits and job security. Conditions such as excessive workloads, short-term contracts, low pay and poor benefits constitute exploitative practices antithetical to decent work. Research on job stress in banking further shows that work intensification and stress sharply undermine satisfaction and retention: Lin et al. (2024) find that higher work-related stress in bank employees is linked to lower job satisfaction and higher turnover intention. These findings mirror our results. Despite formal compliance with work-hour standards, many employees report unsustainable workloads and managerial pressures that violate decent work principles.

Our research findings regarding contested pay further align with evidence that pay equity is critical to decent work. Olubiyi (2023) emphasizes that to achieve Decent Work (SDG 8), Nigerian banks must ensure employees “are well compensated according to industry best practices.” Likewise, Mabaso et al. (2026) found that both pay equity and job equity strongly drive job satisfaction, whereas perceived inequities in compensation or workload undermine motivation and attachment. Our data on unpaid overtime and unfair contracts fit this pattern, as industry reports indicate that a very high percentage of finance workers routinely put in unpaid overtime (e.g., 86% in one survey), and that refusing unpaid overtime is considered career-limiting. These exploitative compensation practices are well-documented as a source of dissatisfaction. In sum, our findings reinforce extant literature showing that unfair pay and workload imbalances (particularly for contract staff) are prevalent in banking and erode the promise of decent work (Mabaso et al., 2026; Olubiyi, 2023; Van der Merwe and Malan, 2020).

#### 5.1.3 Career development: a two-tier system

The findings revealed notable development discrepancies, with temporary workers being systematically excluded from growth opportunities. In stark contrast, permanent employees could access comprehensive development programs. This disparity contradicts fundamental decent work principles of equality and non-discrimination, aligning with Esieboma's (2020) observation about limited prospects for temporary employees.

Existing literature consistently identifies career advancement opportunities as a key component of decent work and employee retention (Ludwig and Webster, 2020). Similarly, participants in the present study highlighted the importance of training, skills development, mentoring, and opportunities for career progression in shaping their perceptions of decent work. Similarly, Lips-Wiersma and Hall (2007) argue that contemporary employees increasingly seek opportunities for continuous learning and career development as part of a meaningful employment experience. In the South African context, Muleya et al. (2022) found that career development opportunities are significant predictors of employee commitment, suggesting that employees are more likely to remain engaged when organizations invest in their professional growth. Furthermore, Houssein et al. (2020) reported a positive relationship between career development and employee retention among financial sector employees, while Alzghoul and Khaddam (2024) found that structured career development initiatives and talent management practices significantly enhance employee retention in banking institutions. The findings of these previous studies support participants' views that access to development opportunities and clear career pathways contribute positively to perceptions of decent work and long-term organizational commitment.

#### 5.1.4 Organizational culture and management practices

The current findings revealed the critical role of organizational culture and management practices in shaping decent work experiences in the financial services institution. This transformation supports Nnaji-Ihedinmah et al.'s (2021) research, which suggests that organizational culture has a significant influence on employee loyalty in the financial services sector. Some participants experienced organizational cultures aligned with decent work principles. These positive experiences align with Shah (2024) finding that organizational culture has a significant impact on employee retention.

Open-door policies emerged as a necessary element of decent work. This supports the International Labour Organisation's (1999) emphasis on social dialogue as being essential for decent work, enabling consultation and negotiation that incorporates workers' perspectives. However, poor management practices undermined the provision of decent work. This finding supports Vilakazi et al.'s (2026) research, which suggests that supportive leadership has a significant impact on employee motivation and retention in financial services, indicating that management behavior can negate otherwise favorable conditions.

### 5.2 Objective 2: to explore the mechanisms that could be adopted to enhance decent work while improving employee retention in the financial services institution

The participants proposed several mechanisms to enhance decent work and to improve employee retention, which translate into clear suggestions by participants for the financial services institution. These suggested mechanisms addressed both immediate practical improvements and fundamental structural reforms that are required to achieve decent work for all employees.

#### 5.2.1 Restructuring pay, rewards and recognition systems

The first key mechanism from the study's findings to enhance decent work and to improve employee retention is the need to fundamentally restructure compensation and benefits to ensure equity between permanent and temporary employees. This suggests that organizations should extend basic benefits such as medical aid, pension contributions, and overtime pay to all employees, regardless of contract type, which aligned with Mackett's call to address persistent labor market disparities.

Furthermore, the participants recommended prioritizing comprehensive benefits over high salaries. This recommendation aligns with Jaiswal's (2019) emphasis on ensuring competitive and equitable compensation based on responsibilities and market norms. Organizations should, therefore, develop transparent compensation frameworks that ensure equal pay for equal work, regardless of employment status.

As part of the mechanisms to enhance decent work and employee retention, the participants further suggested the enhancement of recognition systems as being crucial. This supports Mabaso et al.'s (2026) claim that fair rewards are essential for retention. Democratizing the design of recognition systems was considered by participants as key to enhancing employees' sense of dignity and fairness at work, thus strengthening their commitment to stay. This participatory approach aligns with the International Labour Organisation's (1999) emphasis on social dialogue in workplace decisions.

Non-monetary appreciation was also deemed as essential by participants. This desire for simple acknowledgment aligns with Vilakazi et al.'s (2026) research, which suggests that supportive leadership has a significant impact on retention in the financial services industry, indicating that recognition need not always involve a monetary form.

#### 5.2.2 Extensions of access to development opportunities

Another mechanism that the participants suggested to enhance decent work and to improve employee retention is the extension of development opportunities to all employees. This recommendation directly addresses the systemic exclusion of some categories of workers, as revealed in the study's findings, which aligns with Wang and Cheung's (2024) emphasis on the importance of enhancing employee training and development.

Additionally, the participants suggested focusing on portable, formal qualifications, reflecting the need for development programs that benefit both the organization and individual career security. The suggestion extends to providing AI and digital skills training. This aligns with Nesindande et al.'s (2024) assertion that accelerating investment in employees' skills development is necessary to adjust to emerging trends.

#### 5.2.3 Institutionalization of flexible work arrangements

Another key mechanism suggested by the participants to enhance decent work and to improve employee retention is the institutionalization of flexible work arrangements. This finding corroborates Wang and Cheung's (2024) claim that employees value organizations prioritizing their overall wellbeing. However, according to BambooHR's (2024) Return to Office survey, some employees may prefer to work from the office owing to home-based challenges, with 11% citing too many distractions in their home offices, 13% reporting inadequate equipment, and others mentioning family disruptions such as children at home who interfere with their work productivity. Additionally, Sutarto et al. (2022) found that key challenges for remote workers include a lack of technical equipment, overlapping of work and personal lives, and unstable internet connections, which are particularly relevant in contexts experiencing infrastructure challenges.

Participants further asserted that the institutionalization of flexible work arrangements demonstrates respect for employees' personal lives while maintaining productivity. These recommendations support Wang and Cheung's (2024) observation that employees value organizations that prioritize their overall wellbeing. Organizations should, therefore, formalize flexible work policies that apply equitably to all employees, and not merely senior or permanent staff.

## 6 Practical implications based on the research findings

The findings offer practical insights for financial services organizations aiming to improve employee retention through the promotion of decent work principles. Based on the research findings, the authors propose the following recommendations.

The financial services institution should fundamentally reform its employment structures to remove the divide between permanent and temporary employees, which often undermines the notion of decent work more on the part of temporary employees. This may be achieved by creating clear pathways and defined timeframes, such as transitioning temporary employees to permanent roles within a maximum of 12 months. Organizations should also ensure that essential benefits, including medical aid, pension contributions, and overtime compensation, are extended to all employees irrespective of employment status. Such structural reform is critical, as other interventions are unlikely to succeed in the presence of systemic inequality. Furthermore, regulatory authorities should impose stricter controls on the use of temporary contracts for roles that are inherently permanent, thereby limiting exploitative employment practices.

Equitable access to training and development initiatives should be ensured for all employees, regardless of their contractual status. Organizations are encouraged to offer study support and bursary schemes to long-term temporary employees, recognizing the mutual benefits of such investment. This should include targeted training in digital and AI-related competencies to address technological advancements within the sector. Development initiatives should prioritize formal, transferable qualifications that enhance both organizational capacity and individual career security. In addition, mentorship programs and career guidance services should be made available to support employees' professional growth.

A lack of transparency in remuneration practices erodes trust and undermines decent work. The financial services institution should therefore implement clear and transparent salary structures, along with defined progression criteria, enabling employees to understand how their earnings align with both market standards and internal benchmarks. Adopting role-based, rather than individual-based, remuneration systems can further promote the principle of equal pay for equal work. Organizations should also introduce inclusive recognition programs that acknowledge both major accomplishments and everyday contributions, ensuring that temporary employees are equally recognized. Regular salary reviews should be conducted to maintain fairness and competitiveness.

Flexible working arrangements have demonstrated benefits for both organizational performance and employee wellbeing. Organizations should formalize hybrid work policies that are applied equitably across all employee categories, rather than being limited to senior or permanent staff. This includes providing the necessary technological resources to support effective remote work. Flexibility can enhance work–life balance and reduce commuting costs, which is particularly relevant in the South African context. However, implementation should account for challenges such as the digital divide, load shedding, and unreliable internet access, to avoid disadvantaging certain groups. Clear guidelines regarding eligibility and expectations should be established, allowing employees a degree of choice while acknowledging diverse working preferences.

Meaningful cultural transformation requires alignment between organizational values and structural practices. Organizations should ensure that narratives of inclusiveness are supported by practices that treat all employees with fairness, dignity, and respect. This includes equipping managers with inclusive leadership skills that recognize the contributions of all employees, irrespective of contract type. Creating psychologically safe environments is essential, enabling employees to raise concerns without fear of negative repercussions, such as nonrenewal of contracts. Regular organizational culture assessments should be conducted to evaluate whether espoused values align with employees' lived experiences. Additionally, feedback mechanisms should be inclusive, ensuring that temporary employees are involved in decision-making processes.

The study highlights job security as a fundamental component underpinning other aspects of decent work. Financial services organizations should therefore promote greater employment stability through longer-term contracts and transparent renewal procedures. During periods of economic uncertainty, efforts should be made to retain employees rather than resorting to retrenchments, recognizing that job security fosters both loyalty and productivity. For roles that are genuinely temporary, clear communication regarding contract duration and potential extensions should be provided from the outset. Furthermore, organizations should implement succession planning strategies and create internal advancement opportunities that prioritize existing temporary employees for permanent roles.

## 7 Limitations of the study and recommendations for future research

The limitations of this study present several avenues for future research. Although the focus on a single organization allowed for an in-depth exploration of experiences within a specific context, it restricts the broader generalizability of the findings across the financial services sector. However, the study retains transferability to other contexts, which is a recognized strength of qualitative case study research. Future studies could include multiple organizations across banking, insurance, and investment sectors to identify potential sector-specific differences in perceptions of decent work and retention drivers.

While the qualitative approach yielded rich, in-depth insights into participants' lived experiences, it does not allow for the measurement of relationships between variables. Quantitative research could be employed to examine the links between dimensions of decent work and retention outcomes, potentially enabling the development of predictive models of employee turnover based on these factors. Alternatively, mixed-methods designs could integrate the depth of qualitative insights with the rigor of statistical analysis.

The cross-sectional nature of the study captures perceptions at a single point in time and does not account for how these may change. Longitudinal research could explore how perceptions of decent work and retention evolve throughout employees' careers, identifying key stages where interventions may be most impactful. Such studies could also assess whether improvements in working conditions lead to sustained retention over time.

Furthermore, the study emphasized individual perceptions without explicitly examining the organizational policies and practices that shaped these experiences. Future research could adopt a multi-level perspective, investigating how organizational strategies, departmental cultures, and individual characteristics interact to influence experiences of decent work and retention decisions. Comparative studies between organizations with high and low turnover rates may also help to identify best practices in fostering decent work environments.

Lastly, while the South African context offers valuable insights relevant to emerging economies, the findings may not fully reflect conditions in developed countries with different labor market dynamics. Cross-national comparative studies could therefore distinguish between universal and context-specific elements of decent work and retention, providing guidance for global financial services organizations operating across diverse environments.

## 8 Conclusion

This study offers meaningful insights into the intricate relationship between perceptions of decent work and employee retention within a South African financial services organization. The observed interplay between perceptions of decent work and retention factors indicates that organizations cannot effectively address retention issues through isolated initiatives. Rather, a holistic approach is required, one that simultaneously considers job security, quality of management, recognition, flexibility, opportunities for development, remuneration, and organizational culture. The positive impact of recognition initiatives and flexible work arrangements suggests that even relatively modest investments in improving working conditions can produce substantial gains in employee retention.

As the financial services sector continues to evolve in response to technological change, shifting workforce expectations, and economic uncertainty, the promotion of decent work conditions becomes increasingly vital for organizational sustainability. Organizations that embed decent work principles within their employment practices are likely to gain a competitive edge through improved retention, higher levels of employee engagement, and more robust organizational cultures.

From a theoretical perspective, this study contributes to the understanding of decent work by illustrating how its application varies across different employment categories, thereby emphasizing the need for inclusive and equitable approaches. Practically, it offers evidence-based recommendations for strengthening retention through comprehensive interventions that address the diverse needs and expectations of employees.

Ultimately, the findings highlight that employee retention within the financial services sector extends beyond competitive remuneration or isolated benefits. It requires a sustained commitment to fostering work environments in which all employees, regardless of employment status, experience security, respect, fair compensation, and opportunities for advancement. By adopting a comprehensive approach to decent work, organizations can cultivate sustainable employment relationships that benefit both employees and overall organizational performance in an increasingly demanding labor market.

## Data Availability

The raw data supporting the conclusions of this article will be made available by the authors, without undue reservation.
